# AAV1.NT-3 gene therapy for X-linked Charcot–Marie–Tooth neuropathy type 1

**DOI:** 10.1038/s41434-021-00231-3

**Published:** 2021-02-04

**Authors:** Burcak Ozes, Morgan Myers, Kyle Moss, Jennifer Mckinney, Alicia Ridgley, Lei Chen, Shasha Bai, Charles K. Abrams, Mona M. Freidin, Jerry R. Mendell, Zarife Sahenk

**Affiliations:** 1 Center for Gene Therapy, The Abigail Wexner Research Institute at Nationwide Children’s Hospital, Columbus, OH USA; 2 Department of Pediatrics and Neurology, Nationwide Children’s Hospital and The Ohio State University, Columbus, OH USA; 3 Department of Biomedical Informatics, The Ohio State University College of Medicine, Columbus, OH USA; 4 Biostatistics Resource at Nationwide Children’s Hospital, Columbus, OH USA; 5 Department of Neurology and Rehabilitation, University of Illinois at Chicago, Chicago, IL USA; 6 Department of Pathology and Laboratory Medicine, Nationwide Children’s Hospital, Columbus, OH USA

**Keywords:** Gene therapy, Neurological disorders

## Abstract

X-linked Charcot-Marie-Tooth neuropathy (CMTX) is caused by mutations in the gene encoding Gap Junction Protein Beta-1 (GJB1)/Connexin32 (Cx32) in Schwann cells. Neurotrophin-3 (NT-3) is an important autocrine factor supporting Schwann cell survival and differentiation and stimulating axon regeneration and myelination. Improvements in these parameters have been shown previously in a CMT1 model, Trembler^J^ mouse, with NT-3 gene transfer therapy. For this study, scAAV1.tMCK.NT-3 was delivered to the gastrocnemius muscle of 3-month-old Cx32 knockout (KO) mice. Measurable levels of NT-3 were found in the serum at 6-month post gene delivery. The outcome measures included functional, electrophysiological and histological assessments. At 9-months of age, NT-3 treated mice showed no functional decline with normalized compound muscle action potential amplitudes. Myelin thickness and nerve conduction velocity significantly improved compared with untreated cohort. A normalization toward age-matched wildtype histopathological parameters included increased number of Schmidt-Lanterman incisures, and muscle fiber diameter. Collectively, these findings suggest a translational application to CMTX1.

## Introduction

Gap Junction Protein Beta-1 (encoded by *GJB1*), also known as connexin 32 (Cx32), is located on the X-chromosome accounting for X-linked Charcot-Marie-Tooth (CMTX), which represent at least 10% of CMT neuropathies [1, 2]. Manifesting female carriers usually present with a milder phenotype due to random X-inactivation. Male patients often show intermediate slowing of motor nerve conduction velocity (NCV; 25–35 m/s) and females present mildly slowed motor NCVs (>35 m/s) [3]. Nerve biopsies typically show loss of myelinated fibers, regenerating axon clusters, thinly myelinated axons, and occasional onion bulb formations [4, 5]. *GJB1* is expressed in several tissues although its Schwann cell (SC) expression is relevant to the pathology. The protein is localized to the non-compacted myelin of the paranodes and Schmidt- Lanterman incisures (SLIs) of SCs where it is thought to provide reflexive gap junction channels allowing for the exchange ions and small signaling molecules and metabolites [6–9].

Previous studies from our laboratory in the Trembler^J^ (Tr^J^), a mouse model with a point mutation in *PMP22* established the potential therapeutic use of neurotrophin 3 (NT-3) to improve nerve regeneration and myelination [10, 11]. NT-3 is an important autocrine factor supporting SC survival and differentiation in the absence of axons [12]. The prior translational studies showed the potential efficacy of the intramuscular (IM) delivery of the self-complementary (sc)AAV1.tMCK.NT-3 in Tr^J^ mice, producing functional, electrophysiological, and histopathological improvements [11]. This approach ensures continuous systemic availability of NT-3 via transduction of muscle under a muscle specific promoter, tMCK. NT-3 secreted into circulation can be measured using an enzyme-linked immunosorbent assay as previously shown [11]. The systemic efficacy of NT-3 was proven in Tr^J^ mice, providing evidence of histopathological and electrophysiological improvements from contralateral legs, comparable to the treated legs [11]. In the current study, we used the same logic and tested the efficacy of NT-3 gene therapy in the *Gjb1/*Cx32 null (Cx32 KO) mouse model for CMTX1, another model for primary SC genetic defect. Cx32 KO mice develop a progressive demyelinating neuropathy at ~3 months of age with thin myelin sheaths, onion bulb formation, and enlarged periaxonal collars and SLIs [13, 14]. As we observed in the Tr^J^ model [15], nerve segments distal to the crush site showed impaired regeneration with reduced number of myelinated fibers in the regenerating Cx32 KO nerves compared to wild type (WT; unpublished data, Supplementary Fig. S1), justifying the use of this model to investigate the potential use of NT-3 gene therapy for CMTX1. In addition, similar to the findings in Tr^J^ nerves, the Cx32 KO model shows secondary axonal pathology including decreased axonal diameters of large myelinated fibers and decreased phosphorylation of NFs which precedes demyelination [16]. In this report here, we show that NT-3 gene therapy prevented functional decline and improved electrophysiological and histopathological phenotype of Cx32 KO mouse model by intramuscular delivery of scAAV1.tMCK.NT-3.

## Results

### rAAV.NT-3 vector production and potency

scAAV1.tMCK.NT-3 design (Supplementary Fig. S2a) and production followed previously described methods at Nationwide Children’s Hospital, Columbus [11]. scAAV1.tMCK.NT-3, at 1 × 10^11^ vg dose was delivered to the gastrocnemius muscle of Cx32 KO mice at 3 month of age; blood samples from anesthetized treated and untreated mice were collected by cardiac puncture at 6 months post gene injection and serum was assayed for NT-3 levels using a capture ELISA as previously reported [11]. These serum levels (Supplementary Fig. S2b) were correlating with functional and histologic outcome measures as described.

### Efficacy of NT-3 gene transfer in peripheral nerves of Cx32 KO mice post scAAV1.tMCK.NT-3

#### Functional and electrophysiological studies

**Fig. 1:**
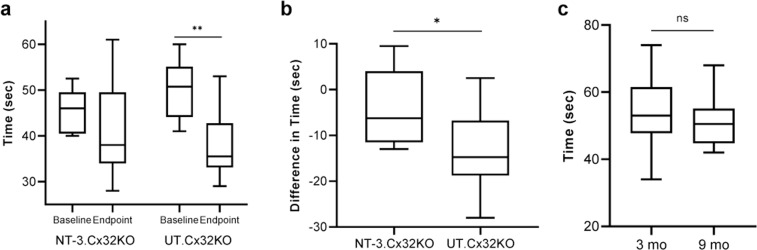
Efficacy of NT-3 gene transfer in Cx32 KO mice on rotarod performance, tested at baseline and 6 months post-treatment. Statistically, there was no difference between baseline and endpoint for the NT-3 group (*n* = 11, *p* = 0.11), while there is a significant reduction in the UT group (*n* = 13, **p = 0.002), Wilcoxon Rank Sum test for paired data (**a**). The difference (or delta reduction) in rotarod test time (endpoint-baseline) by group showed that the UT group had a clinically and statistically larger reduction in rotarod test time than the NT-3 group (median reduction time of 14.75 vs 6.25 s, *P* = 0.026), Wilcoxon Rank Sum test for unpaired data (**b**). Rotarod performance test of 3 (*n* = 13, median, 53 s) and 9 months old (*n* = 10, median, 50.5 s) wild type mice did not show any significant difference. ^ns^*p* > 0.05, unpaired *t*-test (**c**).

Rotarod performance, tested at baseline and 6 months post-treatment showed that gene transfer in Cx32 KO mice preserved function (Median: 46 s. at baseline vs. 38 s. at endpoint; *n* = 11, *p* = 0.11) without significant decline (Fig. 1a), similar to the observations in the age-matched WT mice (Fig. 1c). Comparatively, in the untreated KO controls (Median: 51 s. at baseline vs. 35.5 at endpoint; *n* = 13, *p* = 0.002) there was a significant drop in the rotarod test time in spite of a higher performance level at baseline than the treated group (Fig. 1a).

Difference in rotarod test time was calculated and the UT group showed a clinically and statistically larger reduction in rotarod time than the NT-3 treated group (median reduction time of 14.75 vs 6.25 s, *P* = 0.026, Fig. 1b). There was no difference between male and female performances (Supplementary Fig. S3a).

**Fig. 2:**
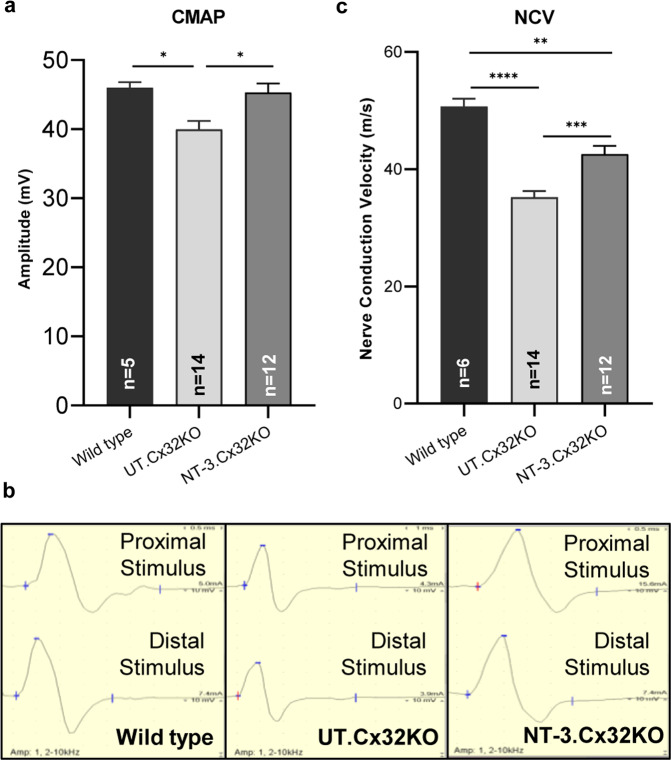
Sciatic nerve conduction studies in Cx32 KO and age matched WT mice at 6 months post NT-3 gene transfer. In Cx32 KO mice, CMAPs were significantly improved with NT-3 gene therapy compared to the untreated (UT) controls. Compared to WT controls there was normalization of the CMAP amplitude while the UT group had 13% smaller CMAP amplitude than the WT mice (**a**). Error bars are ±SEM; *n* = 5 (WT), *n* = 14 (UT), *n* = 12 (NT-3), *p = 0.0307 (WT vs. UT), *p = 0.0103 (UT vs. NT-3), One-way ANOVA with Tukey’s multiple comparisons test. Representative waveforms of the sciatic nerve motor nerve conduction from WT, UT and NT-3 treated Cx32 KO are shown; base time for middle panel is 1 ms while others are 0.5 (**b**). The nerve conduction velocity (NCV) was also significantly improved with treatment corresponding to a 20.9% increase compared to UT cohort (**c**). NCV in the UT mice were 30.5% slower compared to WT whereas this slowing decreased to 16% in NT-3 treated mice. Error bars are ±SEM; *n* = 6 (WT), *n* = 14 (UT), *n* = 12 (NT-3), ****p < 0.0001 (WT vs UT), **p = 0.0016 (WT vs NT-3), ***p = 0.0003 (UT vs NT-3), One-way ANOVA with Tukey’s multiple comparisons test.

Sciatic nerve conduction outcome studies supported efficacy of AAV1.NT-3 gene therapy. Compound muscle action potentials (CMAPs) were significantly improved compared to the untreated controls (AAV1.NT-3: 45.28 ± 1.27 mV, *n* = 12 vs. control KO: 39.96 ± 1.17 mV, *n* = 14, *p* = 0.0103; Fig. 2a, b; Table 1). The NCV and distal latency changes were also significantly improved with treatment (AAV1.NT-3: 42.58 ± 1.32 vs. control KO: 35.21 ± 1.01 m/s; *p* = 0.0003; Fig. 2c, Table 1). Compared to WT controls, we found normalization of CMAP amplitude with treatment while the untreated Cx32 KO mice had 13% smaller CMAP amplitude than the WT mice. Slowing of NCV in the untreated KO mice was about 30.5% compared to WT (*p* < 0.0001), which improved significantly with NT-3 treatment (Fig. 2c, Table 1). There was no significant difference in CMAP and NCV data between sexes (Supplementary Fig. S3b, c).

**Table 1:** Electrophysiological analysis of the sciatic nerve at the end point.

Cohorts	*n*	Latency (ms)	Amp (mV)	CV (m/s)
Untreated	14	0.86 ± 0.03	39.96 ± 1.17	35.21 ± 1.01
NT-3 Treated	12	0.62 ± 0.02****	45.28 ± 1.27**	42.58 ± 1.01****
Wild type	6	0.58 ± 0.03^††††^	45.98 ± 0.72^†^	50.67 ± 1.24^††††^

Data represented as mean ± SEM.

**p ≤ 0.01, ****p ≤ 0.0001 between NT-3 treated and untreated group at the end point.

^†^*p* ≤ 0.05, ^††††^*p* ≤ 0.0001 between WT and untreated group at the end point, One-way ANOVA with Tukey’s multiple comparisons test.

#### Morphological studies

**Fig. 3:**
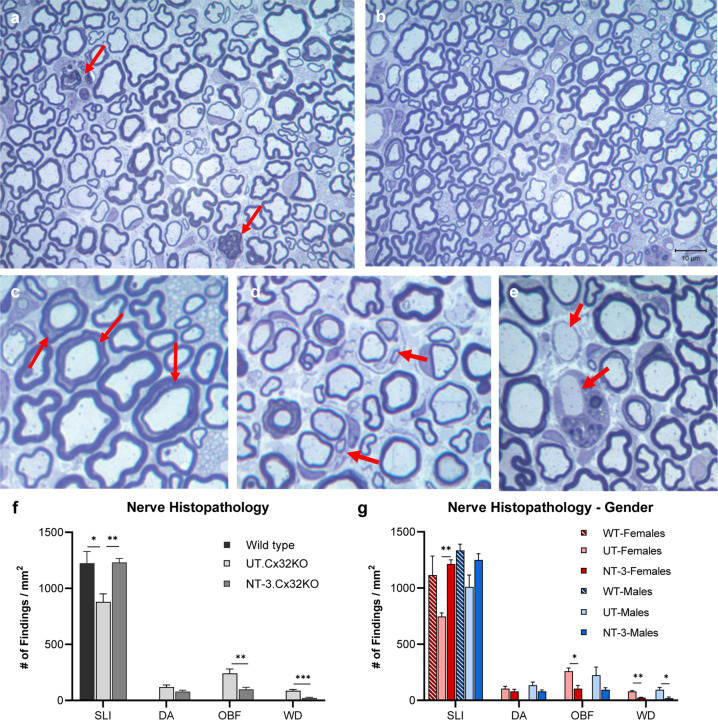
NT-3 gene transfer improves sciatic nerve histopathology in Cx32 KO mice. One-micron-thick, toluidine blue-stained cross-sections of sciatic nerves from untreated and NT-3-treated mice (**a**–**e**) at 6 months post gene transfer. The thinly myelinated axons commonly seen in the untreated nerves (**a**) are decreased with treatment (**b**). Arrows indicate Wallerian degeneration (**a**), Schmidt–Lanterman incisures (**c**) onion bulbs (**d**), and acutely demyelinated axons (**e**). Efficacy of NT-3 gene therapy assessed by quantification of histopathological findings on sciatic nerves from NT-3 treated and untreated (UT) Cx32 KO mice and WT controls (**f**). NT-3 treated mice showed improvements in the frequency of Schmidt–Lanterman incisures (SLI), demyelinated axons (DA), onion bulb formations (OBF), and acute Wallerian degeneration (WD). Increase in the number of SLIs and reduction in OBF and WD profiles were observed predominantly in the females (**g**). Error bars are ±SEM; *n* = 8 per group, *p < 0.05; **p < 0.01; ***p < 0.001; One-way ANOVA with Tukey’s multiple comparisons test for SLI and unpaired *t* test for other findings.

Age-dependent demyelination with evidence of demyelination/remyelination (represented as onion bulbs), adaxonal and incisural (Schmidt-Lanterman) changes, and clusters of regenerating sprouts in the Cx32 KO mice have been previously described in detail at the light and ultrastructural level [14]. In our sciatic nerve samples from untreated KO mice, thinly myelinated and solitary naked axons, onion bulb formations indicative of repeated attempts of remyelination, and profiles of Wallerian degeneration were frequently encountered (Fig. 3a–e). At 6 months post gene transfer, there was a notable increase in the number of myelinated fibers with SLIs while thinly myelinated axons, demyelinated/nude axons, onion bulb formations and profiles of acute Wallerian degeneration appeared less frequently (Fig. 3b). Efficacy of NT-3 gene therapy in the sciatic nerves was quantified by determining the frequency of these histopathological findings (Fig. 3a, c–e). NT-3 treatment resulted in a significant increase in the number of myelinated fibers displaying SLIs on cross sections of sciatic nerves compared to the untreated KO nerves (Fig. 3f). This SLI density increase in the treatment cohort was not different from nerves of age-matched WT mice, suggesting that NT-3 treatment normalized the frequency of SLI formations, which tends to increase with normal aging [17] (Supplementary Fig. S4). With AAV1.NT-3 gene therapy, we observed normalization of SLI densities in both females and males; although interestingly, the mean density in the untreated KO females was lower than treated. The frequency of acute demyelination represented as naked axons, onion bulb formations and Wallerian degeneration profiles were decreased with treatment and the improvements in the latter two parameters were more prominent in females (Fig. 3g).

**Fig. 4:**
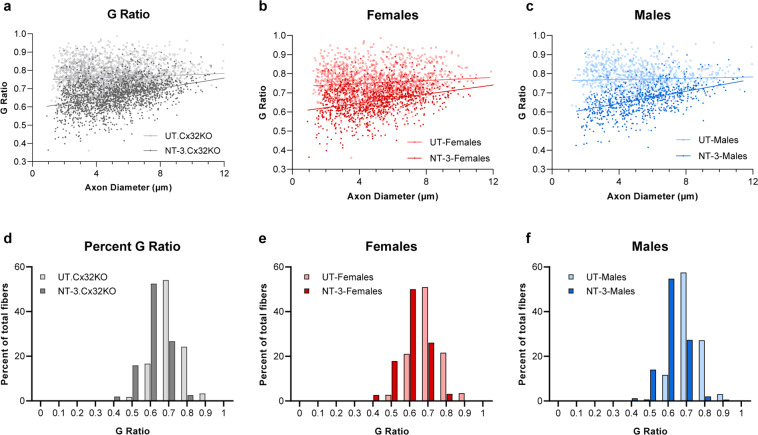
NT-3 gene transfer improves myelin thickness in Cx32 KO peripheral nerves. G ratios calculated for the sciatic nerve of the treated and untreated mice shown as scatterplots against respective axon diameters. Lines indicate linear regression (**a**–**c**). The slopes are significantly different between the two groups, for genders combined (**a**; NT-3, *r*^2^ = 0.150; UT, *r*^2^ = 0.0074; Linear regression, *p* < 0.0001), for females (**b**; NT-3, *r*^2^ = 0.098; UT, *r*^2^ = 0.0106; Linear regression, *p* < 0.0001) and for males (**c**; NT-3, *r*^2^ = 0.206; UT, *r*^2^ = 0.0028; Linear regression, *p* < 0.0001). G-ratios shown as percent distribution indicate a shift to the left with an increase in the number of axons with thicker myelin with NT-3 treatment in genders combined (**d**), in females (**e**) and in males (**f**) without gender influence.

At six months post AAV1.NT-3 injection, g ratio (axon diameter/fiber diameter) of the myelinated fibers in the sciatic nerves showed an increase in myelin thickness corroborating the electrophysiological and functional studies (Fig. 4a, d; Supplementary Table S1). The average g ratio in the Ringer’s lactate treated Cx32 KO mice is 0.761 ± 0.001 (derived from three females and two males), significantly greater than that obtained from our aged-matched WT data (0.66 ± 0.002, *p* < 0.0001), reflecting the presence of thinner myelin in this model. With treatment, the g ratio was significantly reduced (0.663 ± 0.002; *n* = 4, 2 females and 2 males, *p* < 0.0001; Supplementary Table S1) without male or female influence (Fig. 4b, c, e, f). The percent of fibers within a g ratio range of 0.4–0.7 constituted about 70% of total myelinated fibers in the AAV1.NT-3 group (Fig. 4d, f). In contrast, sciatic nerves from untreated males appeared more hypomyelinated than females; the percent of fibers with g ratio < 0.7 was only 12.3% in males while the same population was found two times higher, about 24% in females.

Myelinated fiber size distribution histograms from mid-sciatic nerves of treated and untreated cohorts revealed an increase in the subpopulation of fibers with axon diameters between 2 and 6 µm with treatment (NT-3 cohort: 13,702 ± 231 vs. 12,221 ± 666/mm^2^ in the untreated, *n* = 6 in each cohort, *p* = 0.073; Supplementary Fig. S5). The treatment increased the mean fiber density by about 7% (21,030 ± 197 vs. 19,636 ± 609/mm^2^; *n* = 6 in each cohort, *p* = 0.075) without reaching statistical significance (Supplementary Table S2).

**Fig. 5:**
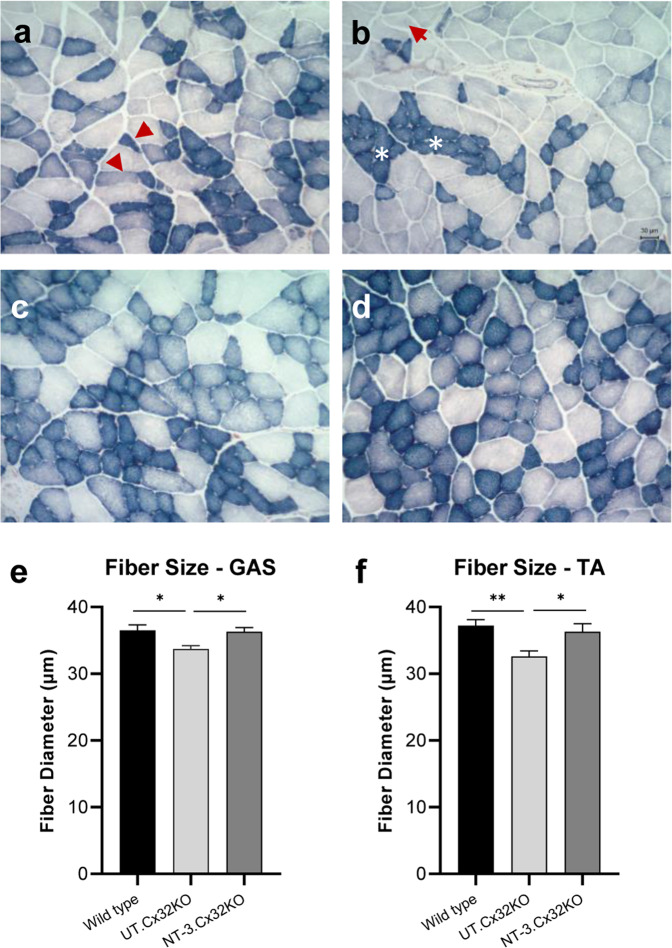
NT-3 gene transfer-induced fiber size increase in Cx32 KO muscle. Representative images of SDH-stained tissue sections from gastrocnemius muscle showing neurogenic changes in the untreated (UT) muscle at 6 months post gene delivery (**a, b**). Slow twitch oxidative (STO, dark), fast twitch oxidative (FTO, intermediate) and fast twitch glycolytic (FTG, light) fibers are seen in the intermediate (**a**) and superficial (**b**) zones from muscle. Arrows indicate small angular fibers of either histochemical fiber types, STO and FTO (**a**) and FTG with STO fiber type groupings marked with asterisks (**b**). Fiber size increases in all fiber subtypes were seen in both females (**c**) and males (**d**). The fiber diameter measurements on gastrocnemius (GAS) and tibialis anterior (TA) muscles showed mean fiber diameters were normalized with NT-3 gene therapy (**e, f**). Error bars are ±SEM; *n* = 8 per group, *p = 0.0155 (WT vs. UT), *p = 0.025 (NT-3 vs. UT) in GAS; **p = 0.0089 (WT vs. UT), *p = 0.0370 (NT-3 vs. UT) in TA, One-way ANOVA with Tukey’s multiple comparisons test.

**Fig. 6:**
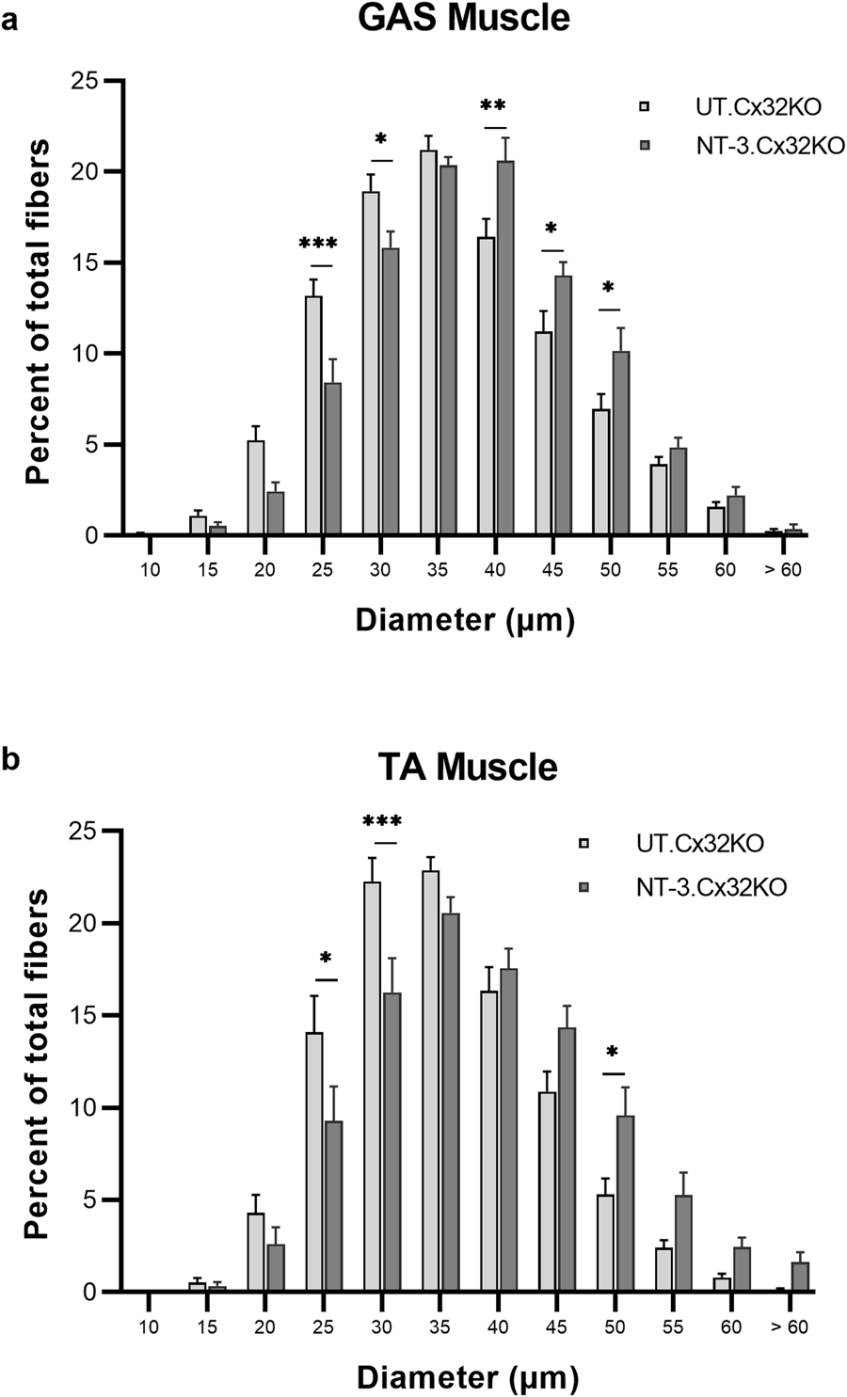
Fiber size distribution histograms of gastrocnemius (GAS) and tibialis anterior (TA) muscles from Cx32 KO mice at six months post NT-3 gene transfer. Size distribution histograms from both muscles showed a shift to larger diameter subgroups with NT-3 gene therapy compared to untreated Cx32 KO cohort (**a b**). *n* = 8 per group. *p ≤ 0.05, **p ≤ 0.01, ***p ≤ 0.001, Bonferroni’s multiple comparisons test.

*Muscle fiber diameter increases at* 6 *months post-treatment:* Gastrocnemius and tibialis anterior muscles from untreated Cx32 KO mice showed scattered or small groups of atrophic fibers belonging to either fast or slow twitch fiber subtypes; this fiber type grouping was compatible with neurogenic change (Fig. 5a, b). AAV1.NT-3 treatment improved the fiber size in both sexes (Fig. 5c, d). We quantified the effects of NT-3 gene therapy in Cx32 KO mice upon muscle fiber size at six months post gene injection in these posterior and anterior compartment muscles of the lower limbs compared to Ringer’s lactate treated KO controls. With treatment, overall diameters in both fatigue resistant slow twitch oxidative and fast twitch fiber subtypes showed significant increases with a mean total fiber diameter (gastrocnemius, NT-3: 36.3 ± 0.5 µm vs. UT: 33.7 ± 0.6 µm, *p* = 0.03; tibialis anterior, NT-3: 36.3 ± 1.2 µm vs. UT: 32.6 ± 0.8 µm, *p* = 0.04, *n* = 8 mice per cohort; Supplementary Table S3),reflecting normalization compared to age matched WT muscle (gastrocnemius, WT: 36.5 ± 0.8 µm vs. NT-3: 36.3 ± 0.5 µm, *p* = 0.97; tibialis anterior, WT: 37.2 ± 0.9 µm vs. NT-3: 36.3 ± 1.2 µm, *p* = 0.79, *n* = 8 mice per cohort) (Fig. 5e, f; Supplementary Table S3). The fiber size difference observed in gastrocnemius muscle between sexes in WT mice was not observed in the Cx32 KO (both treated and untreated, Supplementary Fig. S3d). Similarly, tibialis anterior muscle of WT male was significantly larger than WT female mice. The same difference was also evident in the treated Cx32 KO mice (Supplementary Fig. S3e). The treatment effect was reflected in muscle fiber size distribution histograms from both muscles with a clear shift to right, larger diameter subgroups (Fig. 6a, b). The sex-related differences observed in the total fiber diameter in the tibialis anterior muscle was also reflected in the fiber size distribution, favoring the males towards the larger diameters (Supplementary Fig. S3f). Size increase favoring males was not statistically significant from females in the gastrocnemius muscle (Supplementary Fig. S3g).

## Discussion

We report here proof of principle preclinical studies in support of AAV1.NT-3 gene therapy resulting sustained NT-3 delivery through secretion by muscle cells for the second most common form of CMT neuropathy, CMTX1. scAAV1.tMCK.NT-3 therapy resulted in long term and measurable NT-3 serum levels ensuring systemic effects in the Cx32 KO mice, which in turn led to enduring functional performance, improvements in electrophysiology and histopathology of peripheral nerves, and normalization of muscle fiber size in anterior and posterior compartment leg muscles.

For this experimental paradigm we chose three months of age as onset of treatment when Cx32 KO mice develop histopathological findings [13, 14]. The long-term efficacy of the IM injection of scAAV1.tMCK.NT-3 vector at 1 × 10^11^ vg, was assessed with multiple outcomes. At study termination 6 months post-treatment, serum NT-3 levels were in the nanogram levels with inter-animal variability as previously reported in the Tr^J^ model [11], showing no gender difference. Using a standard rotarod protocol, we showed that NT-3 was protective for maintaining function for at least 6 months of study duration, even though the neuropathy onset preceded gene transfer. We observed no statistical gender difference in rotarod performance of cohorts for treatment effect, although at baseline and endpoint in each cohort, females performed better than males. In contrast, the analysis of electrophysiological studies showing significant improvements with treatment revealed no evidence of gender effect in both treated and untreated cohorts.

It has been reported that the pathological changes in Cx32 KO model are much more robust than either the electrophysiological or the behavioral abnormalities [14]. Our study confirmed these results. By light microscopy, the most striking finding in sciatic nerves from Cx32 KO mice at 9 months of age was the presence of numerous fibers with thinner than normal myelin and occasional demyelinated/nude axons and onion bulb formations. Rare scattered profiles of Wallerian degeneration were also present. In samples from the treatment cohort, there was the expected increase of myelin thickness, however we were surprised with a notable increase in the number of myelinated fibers displaying SLIs. Based on these observations, the quantified histopathological parameters we chose to apply to this study were shown to be sensitive indicators of treatment efficacy. At 6 months post AAV1.NT-3 injection, significantly increased myelin thickness supported by a robust decrease in g ratio correlated with significantly improved sciatic NCV. With improved myelination, the frequency of onion bulb formations decreased and there were fewer occurrences of active demyelination/naked axons. Treatment also resulted in lower frequency of degenerating axons as an indication of improved SC-axon interactions. SLI number is another indication of improved SC-axon interactions with NT-3 gene therapy in this model. With AAV1.NT-3 gene therapy, we observed an increase, in SLI densities compared to untreated mice reaching to WT levels.

It has been known for several decades that the number of incisures per internode increases as internodal width increases in developing, normal adult and regenerated myelinated axons [18]. Studies from our laboratory have shown that in addition to normal aging, disease states can also alter occurrence of SLIs. We found 1.5–1.6-fold increase of SLI densities during regeneration associated myelination within the CMTX1 and CMT1A xenografts [19]. The overproduction of SLIs was reported previously in another model with mutant SCs, in the peripheral myelin of shiverer mice [20]. In contrast, primary axonopathy models may also alter SLI occurrence. As an example, we observed an increase in the number of SLI in a mouse model in which neuron specific over expression of mutant HSPB1 caused age-related motor neuronopathy/axonopathy [17]. These observations lead us to suggest that an increase in the SLI number is likely to be a compensatory mechanism in response to increased metabolic needs for improving efficiency of SC-axon interactions in these conditions [19, 21]. Although in CMTX1 xenografts an increase of SLI density was observed during regeneration associated myelination, CX32 null-SCs failed to show SLI density increase with age. Further studies are needed if this failure is unique to Cx32 KO status or may occur in other models harboring mutant SCs.

The management for CMT symptoms is currently supportive; and the search for treatment points towards gene therapy strategies to replace the defective gene is currently in exploration. In Gjb1/Cx32 knockout (Cx32 KO) mice, the animal model for CMTX, intrathecal injection of a lentivirus expressing *GJB*1 using a SC specific promoter resulted in cell specific expression of Cx32 up to 50% of SCs in throughout the length of the sciatic nerve and improvement in the neurophysiological, neuropathological and motor phenotype of GJB1 null mice [22]. Authors proposed diffusion of the virus from the spinal fluid to the endoneurium of the roots, and subsequently peripheral nerves as likely explanation for SC transduction. Translational validity of this approach, however, remains challenging. Gjb1 was transferred with a lentivirus that can lead to genome integration and insertional mutagenesis. In addition, the dense connective sheath of peripheral nerves in humans could present a barrier to transduction efficiency.

The significance of our study is that AAV1.NT-3 gene therapy, administered after the onset of neuropathy leads to meaningful improvements in functional, electrophysiological and histopathological phenotype of Cx32 KO model for CMTX1, another CMT subtype with primary SC genetic defect, similar to CMT1A. Our proof of principle studies in the mouse model for CMTX along with previously demonstrated safety and efficacy of our method [11] provide a potential path for clinical translation.

## Materials and methods

### Animals, procedures, and treatment groups

The generation and initial characterization of c*x32*- null (*cx32*^−/−^ female and *cx32*^Y/−^) mice has been described [23]. Animals for this study were generated from our colony at The Abigail Wexner Research Institute at Nationwide Children’s Hospital from two breeding pairs of *cx32*-null mice (Cx32^−/−^ females and Cx32^−/Y^ males) obtained from University of Illinois (Dr. C. Abrams). Genotypes were established by PCR analysis of genomic DNA isolated from tail clips. WT mice used in this study were strain-matched to Cx32 KO mice which were generated in C57BL/6 strain. WT breeding pairs obtained from The Jackson Laboratory (strain: 000664) were used for in-house breeding protocol. All animal experiments were performed according to the guidelines approved by The Research Institute at Nationwide Children’s Hospital Animal Care and Use Committee that operates full accordance with the Animal Welfare Act and the Health Research Extension Act (IACUC approval number = AR18-00076). The design of the experimental groups comparing scAAV1.NT-3 vector, dose, and treatment duration is outlined below:

Three-month-old Cx32 KO mice were injected in the right gastrocnemius muscle with either Ringer’s lactate (seven males and seven females, *n* = 14) or 1 × 10^11^ vg of scAAV1.tMCK.NT-3 vector (six males and six females, *n* = 12). Functional status of mice was monitored using rotarod at baseline and endpoint and following electrophysiology mice were euthanized by an over-dosage of xylazine/ketamine anesthesia for harvesting blood, sciatic nerves, and distal leg muscles at 6 months post gene injection.

### rAAV.NT-3 vector production and potency

Design of scAAV vector with serotype 1 containing NT-3 under muscle specific tMCK promoter was described previously and produced in our Viral Vector Core at Nationwide Children’s Hospital, Columbus [11]. Aliquots of virus were kept at −80 °C until used. Blood samples from anesthetized treated and untreated mice were collected by cardiac puncture at six months post gene injection and serum was assayed for NT-3 levels using a capture ELISA as previously reported [11].

### Rotarod testing

Mouse motor function and balance was tested at baseline and endpoint by using the accelerating rotarod (Columbus Instruments, OH, USA) [11]. Mice were trained on the rotarod apparatus for 2 weeks to acclimate to testing protocol prior to data collection. The protocol was run at 5 rpm with a constant acceleration of 0.5 rpm/s, and the average of the best two out of three trials was included.

### Nerve conduction studies

The animals were anaesthetized under 2% isoflurane and heating pad was set to 37 degrees Celsius to maintain body temperature. Right sciatic nerve conduction studies were performed using a Nicolet Viasys Viking Select EMG EP System (Nicolet Biomedical, Wisconsin, USA) and 27G disposable subdermal needle electrodes for both stimulation and recording as described previously [24]. The stimulating electrodes were placed subcutaneously proximal (stimulus 1) and distal side (stimulus 2) of the sciatic notch with a distance of ~2 cm between the electrodes. The recording electrode was placed subcutaneously aligned with the long axis of gastrocnemius muscle and a reference electrode was inserted subcutaneously next to the Achilles tendon at a 30-degree angle, leaving 2–5 mm of the needle under the skin. Latency, CMAP amplitude, area, duration, and nerve conduction velocity were determined. The distance between the two stimulation sites was used to determine the nerve conduction velocity.

### Processing of sciatic nerve for histopathological analysis

The sciatic nerves were removed under a dissecting microscope, fixed in glutaraldehyde; tissue blocks were processed for plastic embedding for light and electron microscopy using standard methods established in our laboratory [25]. Samples were selected for morphometric analysis based on the suitability of the tissue sections, including staining quality, contrast, and lack of artifacts such as wrinkles in the section, and not based on outcomes of behavioral or physiological analyses.

### Myelinated fiber density determinations

Quantitative analysis was performed on 1 µm-thick toluidine blue stained cross sections from mid sciatic nerve segments from treated and untreated cohorts (*n* = 6; 3 males and 3 females in each group). Five randomly selected areas (one from center and four from each quadrants) were photographed at 100× magnification and axon diameter measurements were obtained from the computer screen image frames using BioQuant Life Sciences imaging software (2014, V15.5.6; BioQuant Image Analysis Corporation; Nashville, TN). An average of 1056 measurements per treated and 986 measurements per untreated mouse were made. Composites of fiber size distribution histograms and mean myelinated fiber densities (mean + SEM, number/mm^2^) were generated. In addition, in all stored images from treated and untreated groups (*n* = 8; 4 males and 4 females in each group) myelin profiles with putative SLI (i.e., with a clear circular band separating two dark myelin bands [17, 20], demyelinated/nude axons, onion bulb formations and profiles of acute Wallerian degeneration were identified and the density histograms of these pathological measures were generated as number per total unit area analyzed (0.0502 mm^2^ of endoneuria area per mouse). Measurements from age-matched WT mice were also generated for comparison.

### G ratio of the myelinated fibers

The g ratio refers to axonal diameter/fiber diameter and lower g ratios represent axons with thicker myelin [26, 27]. Measurements were done by outlining the myelin interior and exteriors in Axiovision (AxioVs40x64 V 4.9.1.0) to determine area, which was used to derive diameters to yield g-ratio, similar to the methods used in MRI estimation [28, 29].

For each animal, measurements from all myelinated fibers in three random images (photographed at ×100 magnification) from mid-sciatic nerves were included; a total of 2858 measurements per untreated (three females and two males) and 2167 per scAAV1.tMCK.NT-3 injected cohorts (two females and two males) were obtained to generate the percent g ratio distribution histograms and scattergrams. Slopes of AAV.NT3 treated vs untreated were compared using Graphpad software (Graphpad Prism 8.2.0) [11].

### Histological analysis of muscle

Gastrocnemius and tibialis anterior muscles from scAAV1.tMCK.NT-3 and Ringer’s lactate injected Cx32 KO mice (*n* = 8, equal number of males and females in each group) were removed and 12 µm thick cross cryostat-sections were subjected to succinic dehydrogenase (HDS) enzyme histochemistry to assess fiber type differentiation using standard protocol established in our laboratory for muscle fiber type specific diameter measurements [30, 31]. Briefly, three images, each representing three different zones of muscle (deep, intermediate and superficial) were photographed at ×20 magnification using an Olympus BX41 microscope and SPOT camera (0.2434 mm^2^ area per section, per zone; 0.7302 mm^2^ area per animal). This approach was chosen to capture the alterations in the oxidative state of fibers in each zone in response to treatment. Samples were selected for morphometric analysis based on the suitability of the tissue sections, including staining quality, contrast, and lack of artifacts such as wrinkles in the section, and not based on outcomes of behavioral or physiological analyses. Diameters of fatigue resistant slow twitch oxidative (STO, dark), fast twitch oxidative (FTO, intermediate) and fast twitch glycolytic (FTG, light) fibers were determined by measuring the shortest distance across the muscle fiber using Zeiss Axiovision LE4 software and expressed as percent of total. The mean fiber diameter (mean ± SEM) was derived from combining all three fiber types in each cohort (*n* = 8; 4 males and 4 females from AAV1.NT-3 and untreated Ringer’s lactate injected groups). A total of 2688–2732 fibers for treated mice and 2797–2830 fibers for untreated mice were measured for gastroc and tibialis anterior muscles, respectively, and fiber size distribution histograms were generated.

### Statistics

Adequate sample size was determined according to our previous studies that performed analogous experiments [11, 32, 33]. For comparisons between treated and non-treated groups, statistical analyses were performed in Graph pad Prism 8.2 software. Two tail Student *t*-test, one-way ANOVA with Tukey’s multiple comparison test, Bonferroni’s multiple comparisons test or linear regression analysis were performed based on the data, and significance level was set at *P* ≤ 0.05. The tests that meet the best assumptions of the data were chosen. The variance between the groups that are being statistically compared was similar. Results were given as mean ± SEM in all experiments and the number of animals was mentioned in figure legends along with the name of the statistical analysis performed. Other than the functional and electrophysiology tests, no blinding was used.

## Supplementary information

Supplementary Data-AAV1.NT-3 gene therapy for X-linked Charcot-Marie-Tooth Neuropathy type 1
