# Supplementary material for: AAV1.NT-3 gene therapy for X-linked Charcot–Marie–Tooth neuropathy type 1
Source: Gene Ther. 2021 Feb 4;29(3-4):127–37. doi: 10.1038/s41434-021-00231-3 (PMC9013664; doi:10.1038/s41434-021-00231-3)
Supplement: Supplementary file 1 — Supplementary Data-AAV1.NT-3 gene therapy for X-linked Charcot-Marie-Tooth Neuropathy type 1 [file 41434_2021_231_MOESM1_ESM.pdf]

AAV1.NT-3 gene therapy for X-linked Charcot-Marie-Tooth Neuropathy type 1

Burcak Ozes[1], Morgan Myers[1], Kyle Moss[1], Jennifer Mckinney[1], Alicia Ridgley[1], Lei Chen[1], Shasha Bai[2,3], Charles K. Abrams[4], Mona M. Freidin[4], Jerry R. Mendell[1,5], Zarife Sahenk[1,5,6]

1 Center for Gene Therapy, The Abigail Wexner Research Institute at Nationwide Children's Hospital, Columbus, Ohio
2 Department of Biomedical Informatics, The Ohio State University College of Medicine
3 Biostatistics Resource at Nationwide Children's Hospital
4 Department of Neurology and Rehabilitation, University of Illinois at Chicago
5 Department of Pediatrics and Neurology, Nationwide Children's Hospital and The Ohio State University
6 Department of Pathology and Laboratory Medicine, Nationwide Children's Hospital, Columbus, Ohio

Corresponding author:
Zarife Sahenk, MD, PhD.
Director, Neuromuscular Pathology
700 Children's Drive Rm WA 3024
Tel: 614-722-2875
Fax: 614-722-3273
Email: zarife.sahenk@nationwidechildrens.org

Supplementary Data

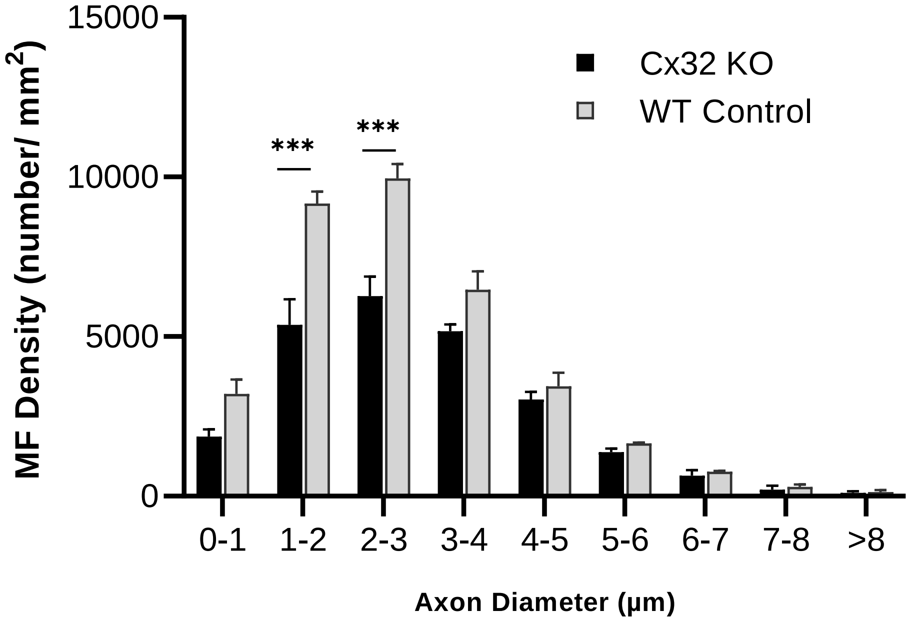

Supplementary Figure 1. Myelinated fiber-axon size distribution in the regenerating sciatic nerves, 5 mm distal to crush site from Cx32 KO and wild type control mice at 2 months post-crush. Small myelinated fiber subpopulation with axon size within 1-2 μm and 2-3 in diameter range was significantly less the Cx32 KO regenerating nerves. The total number of myelinated fiber density (Cx32 KO: 24026.8 ± 1548.1 vs. WT: 35095.1 ± 1283.3; $p<0.0001$) is markedly reduced in Cx32 KO compared to WT regenerating nerves. Error bars represent ± SEM; ***$p\leq 0.001$, ****$p\leq 0.0001$, n=3 per group; Bonferroni's multiple comparisons test.

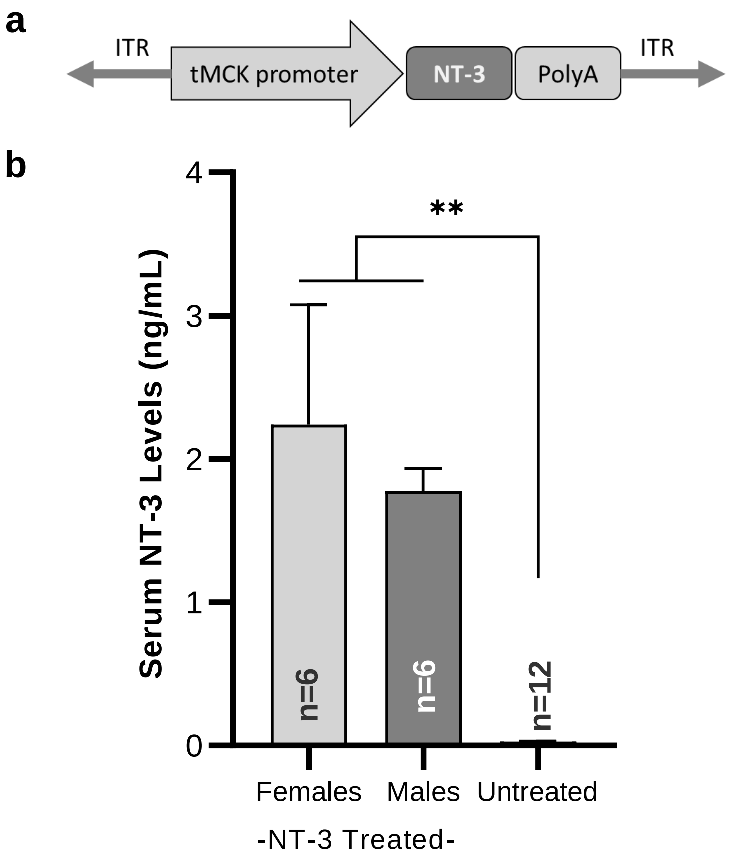

Supplementary Figure 2. scAAV1.tMCK.NT-3 vector and NT-3 serum levels. (a) NT-3 expression is driven by a tMCK in the scAAV vector backbone. The diagram shows the cassette composed of a tMCK enhancer/ promoter region (714 bp), the full-length NT-3 cDNA (774 bp) and the SV40 polyA tail (211 bp). (b) Cx32 KO mice were injected with 1 × 10^{11} vg of scAAV1.tMCK.NT-3 into the right gastrocnemius muscles or Ringer's lactate as control. Serum samples were obtained from treated and untreated Cx32 KO mice via cardiac puncture at six months post-treatment/endpoint, and NT-3 levels were determined by using enzyme-linked immunosorbent assay; shown separately in females and males. Error bars are ± SEM; n=12 (NT-3 treated), n=12 (UT), **P< 0.01; unpaired t test.

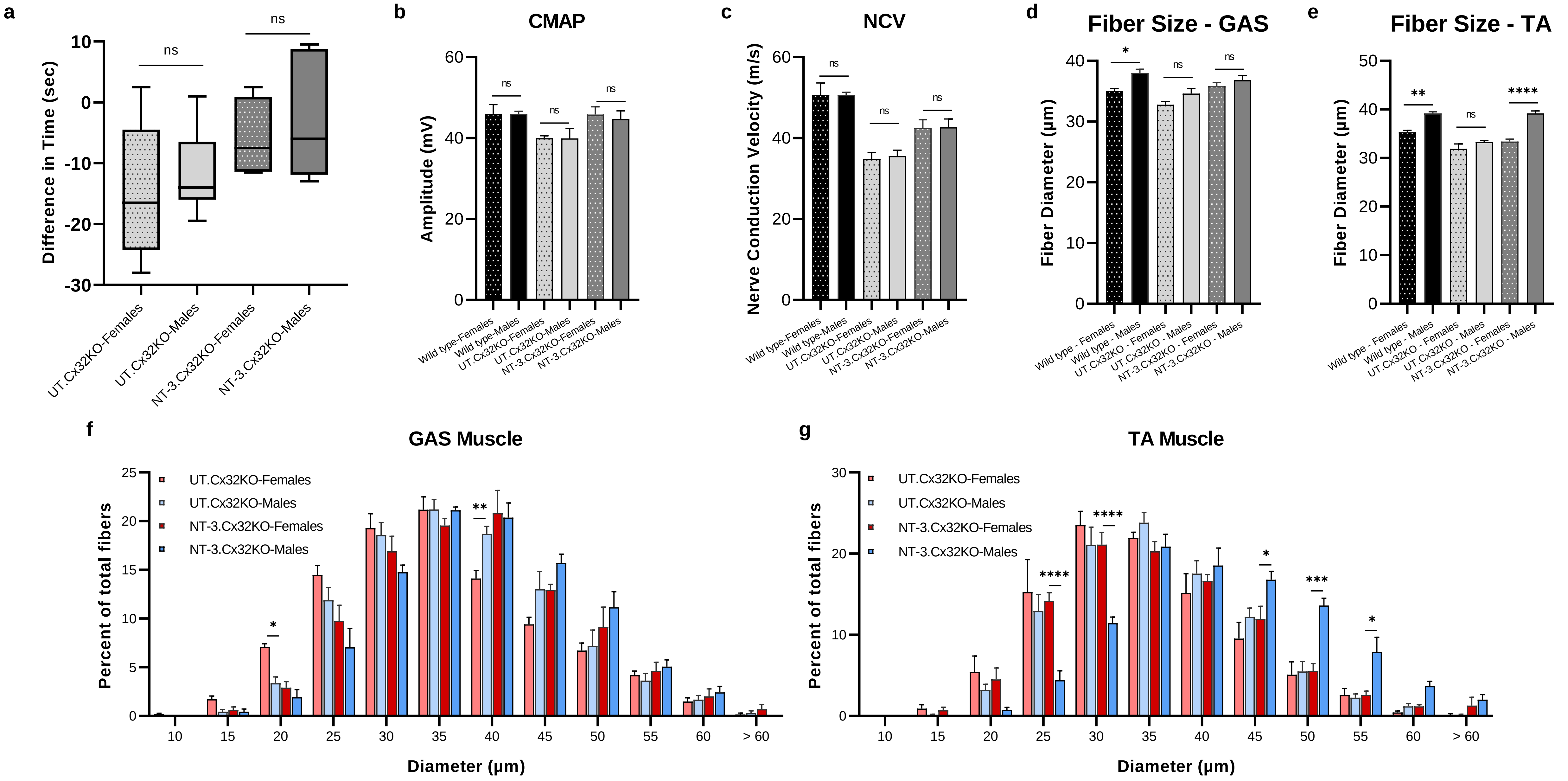

Supplementary Figure 3. The graphs showing analyses on rotarod (a), electrophysiology (b,c), muscle fiber size (d,e) and muscle fiber size distribution (f,g) separated by sex. Error bars represent ± SEM; [ns] p>0.05, *p≤0.05, **p≤0.01, ***p≤0.001, ****p≤0.0001, Tukey's multiple comparisons test.

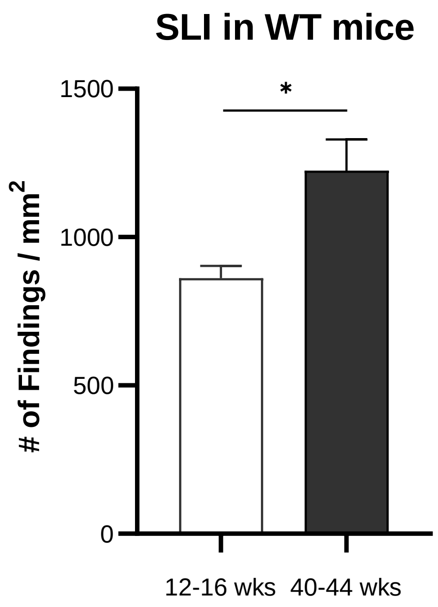

Supplementary Figure 4. Histopathological quantitative analysis of Schmidt-Lanterman incisures (SLIs) performed on sciatic nerves of young (12-16 wks) and old (40-44 wks) wild type mice. Old cohort shows an increase in the number of SLI compared to young mice. Error bars represent ± SEM; *p=0.031, n=4 per group; unpaired t test.

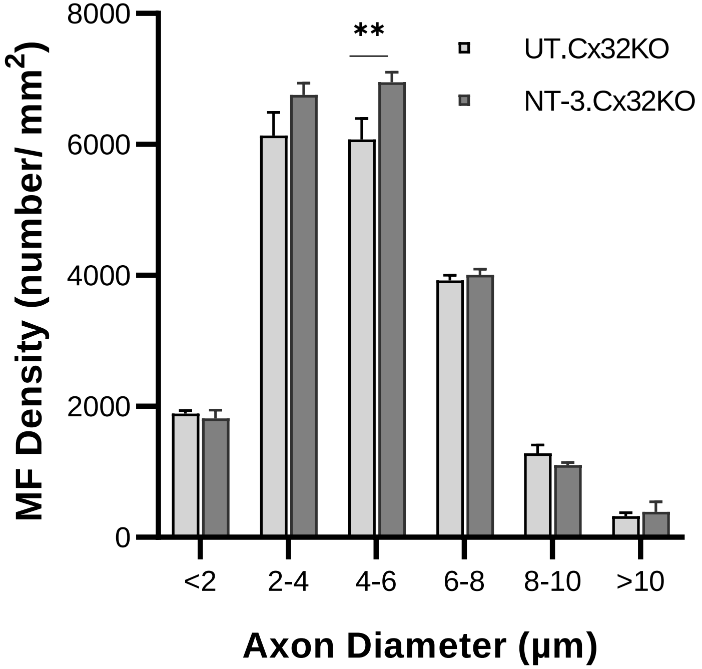

Supplementary Figure 5. Myelinated fiber distribution in the sciatic nerves from NT-3 treated and untreated Cx32 mice. An increase in the subpopulation of axons 2-6 μm in diameter was seen with treatment. Error bars represent ± SEM; **p=0.0046, n=6 per group; Bonferroni's multiple comparisons test.

Supplementary Table 1 Average G ratios of the sciatic nerves from Cx32KO mice

Cohorts	*n*	Number of measurements	Mean ± SEM
Untreated	5	2858	0.761 ± 0.001
NT-3 Treated	4	2167	0.663 ± 0.002****
Untreated-Female	3	1503	0.752 ± 0.002
NT-3-Female	2	1040	0.658 ± 0.002****
Untreated-Male	2	1355	0.771 ± 0.002
NT-3-Male	2	1127	0.667 ± 0.002****

****$p \leq 0.0001$ between NT-3 treated and untreated, unpaired t test

Supplementary Table 2 Myelinated fiber density (number/mm^2) in the sciatic nerves from Cx32KO and wild type mice

**Cohorts**	***n***	**Mean ± SEM**
Untreated	6	19,636 ± 609
NT-3	6	21,030 ± 197
WT (age-matched)	4	20,249 ± 356
WT (young/12-16 wks)	3	21,071 ± 267

Supplementary Table 3 Mean muscle fiber diameter in gastrocnemius and tibialis anterior samples from Cx32 KO and WT mice

Fiber Type	Gastrocnemius					
	Wild type (n=8)		Untreated (n=8)		NT-3 Treated (n=8)	
	Number	Diameter (μm)	Number	Diameter (μm)	Number	Diameter (μm)
STO	101.9 ± 6.8	30.0 ± 0.7††	79.1 ± 6.1	26.4 ± 0.6	75.9 ± 6.2	29.3 ± 0.8*
FTO	48.5 ± 2.6	34.1 ± 0.7††	91.3 ± 4.5	30.7 ± 0.6	77.5 ± 6.2	34.0 ± 1.0**
FTG	170.5 ± 6.3	41.2 ± 1.0†	178.8 ± 8.8	38.5 ± 0.6	182.6 ± 8.6	40.2 ± 0.6
All fiber	320.9 ± 9.7	36.5 ± 0.8†	349.1 ± 6.0	33.7 ± 0.6	336.0 ± 10.4	36.3 ± 0.5*

Fiber Type	Tibialis anterior					
	Wild type (n=8)		Untreated (n=8)		NT-3 Treated (n=8)	
	Number	Diameter (μm)	Number	Diameter (μm)	Number	Diameter (μm)
STO	91.6 ± 10.1	30.5 ± 1.0††	90.6 ± 7.2	25.7 ± 0.6	88.6 ± 11.0	27.9 ± 1.2
FTO	66.3 ± 3.5	36.3 ± 1.1††	95.6 ± 7.5	32.1 ± 0.9	92.5 ± 7.8	35.2 ± 1.2
FTG	162.5 ± 5.7	41.5 ± 0.7††	167.5 ± 6.3	36.6 ± 0.7	160.4 ± 9.6	41.2 ± 1.0**
All fiber	320.4 ± 13.6	37.2 ± 0.9††	353.8 ± 14.7	32.6 ± 0.8	341.5 ± 19.2	36.3 ± 1.2*

Data represented as mean ± SEM
*$p \leq 0.05$, **$p \leq 0.01$ between NT-3 treated and untreated group at the end point
†$p \leq 0.05$, ††$p \leq 0.01$, †††$p \leq 0.001$ between WT and untreated group at the end point, Tukey's multiple comparisons test.
